# Differential Response of Hippocampal Subregions to Stress and Learning

**DOI:** 10.1371/journal.pone.0053126

**Published:** 2012-12-28

**Authors:** Darby F. Hawley, Kristin Morch, Brian R. Christie, J. Leigh Leasure

**Affiliations:** 1 Department of Psychology, University of Houston, Houston, Texas, United States of America; 2 Division of Medical Sciences, University of Victoria, Victoria, British Columbia, Canada; 3 Island Medical Program, University of British Columbia, Victoria, British Columbia, Canada; 4 Department of Biology & Biochemistry, University of Houston, Houston, Texas, United States of America; Max Planck Institute of Psychiatry, Germany

## Abstract

The hippocampus has two functionally distinct subregions–the dorsal portion, primarily associated with spatial navigation, and the ventral portion, primarily associated with anxiety. In a prior study of chronic unpredictable stress (CUS) in rodents, we found that it selectively enhanced cellular plasticity in the dorsal hippocampal subregion while negatively impacting it in the ventral. In the present study, we determined whether this adaptive plasticity in the dorsal subregion would confer CUS rats an advantage in a spatial task–the radial arm water maze (RAWM). RAWM exposure is both stressful and requires spatial navigation, and therefore places demands simultaneously upon both hippocampal subregions. Therefore, we used Western blotting to investigate differential expression of plasticity-associated proteins (brain derived neurotrophic factor [BDNF], proBDNF and postsynaptic density-95 [PSD-95]) in the dorsal and ventral subregions following RAWM exposure. Lastly, we used unbiased stereology to compare the effects of CUS on proliferation, survival and neuronal differentiation of cells in the dorsal and ventral hippocampal subregions. We found that CUS and exposure to the RAWM both increased corticosterone, indicating that both are stressful; nevertheless, CUS animals had significantly better long-term spatial memory. We also observed a subregion-specific pattern of protein expression following RAWM, with proBDNF increased in the dorsal and decreased in the ventral subregion, while PSD-95 was selectively upregulated in the ventral. Finally, consistent with our previous study, we found that CUS most negatively affected neurogenesis in the ventral (compared to the dorsal) subregion. Taken together, our data support a dual role for the hippocampus in stressful experiences, with the more resilient dorsal portion undergoing adaptive plasticity (perhaps to facilitate escape from or neutralization of the stressor), and the ventral portion involved in affective responses.

## Introduction

The hippocampus is a functionally complex brain area that plays a role in behaviors as diverse as spatial navigation and emotion. Not surprisingly then, it is also structurally complex and there is mounting evidence that distinct subregions along it’s longitudinal axis are subservient to different behaviors. The dorsal (septal) component has been linked to spatial navigation [1]–[3], whereas the ventral (temporal) portion has been associated with emotional responses to arousing stimuli [4], [5].

The hippocampus is also particularly sensitive to stress [6], but it appears that the two subregions respond differentially to stressful experiences. For example, acute stressors decrease long term potentiation (LTP) in the dorsal hippocampus, but selectively increase monoamine levels [7] and long-term potentiation in the ventral subregion [8]. Chronic stressors also elicit subregion-specific responses. We have previously shown that adaptive plasticity, such as expression of neuropeptide Y (NPY) and ΔFosB, were highest in the dorsal subregion following chronic unpredictable stress (CUS), whereas adverse events, including decreased survival of hippocampal progenitor cells, were most severe in the ventral subregion [9]. These data suggest that the hippocampus plays a dual role in the response to stress, with the dorsal portion undergoing adaptive plasticity, perhaps to facilitate escape or avoidance of the stressor, and the ventral portion involved in the affective facets of the experience [9]. We reasoned, therefore, that if chronic stress selectively induces adaptive neuroplastic responses in the dorsal hippocampus, spatial navigation would be enhanced by CUS. Accordingly, in the present study, we determined whether CUS enhanced spatial performance in the radial arm water maze (RAWM).

The RAWM is a spatial navigation task that is stressful to laboratory rodents because it involves swimming [10]. It is therefore a suitable means by which to place demands on both hippocampal subregions simultaneously. Spatial learning has previously been associated with increased neurotrophin expression and synaptic remodeling in the hippocampus [11], but whether this varies by subregion has not been investigated. In the present study, we assessed subregion-specific changes in the expression of proteins associated with plasticity, including BDNF, its immature isoform, proBDNF, and postsynaptic density-95 (PSD-95), following a one-day learning paradigm in the RAWM. We hypothesized that protein expression would be higher in the dorsal subregion due to the demands of spatial navigation, and lower in the ventral subregion due to the stressful nature of the learning task.

Finally, the dentate gyrus (DG) of the hippocampus is a neurogenic region, and the generation of neurons along its rostrocaudal extent has been linked to both spatial function [12] and the affective response to stressful experiences [13], [14]. Stress depletes the pool of newly generated cells in the DG [15]. We have shown that this suppressive effect on survival of newborn cells is most severe in the ventral, compared to the dorsal subregion following CUS [9]. In the present study, we extended this finding by also examining proliferation and neuronal differentiation of cells in the dorsal and ventral DG following CUS.

The present study was designed to accomplish three goals. First, we tested the hypothesis that CUS would enhance spatial performance. Second, we examined subregion-specific protein expression after RAWM exposure, which was simultaneously stressful and demanded spatial function. Third, we extended our prior finding that the suppressive effect of CUS on hippocampal neurogenesis is most severe in the ventral subregion. Our results are consistent with the idea that the hippocampus plays a dual role in stressful experiences, with the dorsal subregion selectively involved in adaptive behaviors, and the ventral subserving the emotional response.

## Materials and Methods

### Ethics Statement

All experimental procedures were conducted in accordance with the Guide for the Care and Use of Laboratory Animals of the National Institutes of Health. The relevant animal protocol was approved by the University of Houston Institutional Animal Care and Use Committee (protocol number 10–039).

### Animals and CUS Paradigm

Adult male Long Evans rats (3 months old at the start of experiments) were individually housed in clear plastic cages with *ad libitum* food and water. Upon arrival, animals habituated for one week to the vivarium environment. CUS was administered as previously described [9], [16] for 14 days. Briefly, two different daily stressors (e.g., tilted cages, vinegar-laced water, exposure to strobe light, predator odor and predator calls) as well as the timing of the stressors, were determined by a random number generator. All stressors were conducted in a room separate from where control animals were housed.

### Administration of Thymidine Analogs

In order to quantify the impact of CUS on survival of progenitor cells in the DG, control (n = 9) and stressed (n = 9) animals were injected with iododeoxyuridine (IdU, MP Biomedicals, OH, USA, 57.5 mg/kg, i.p.) daily for the first 5 days of CUS. To quantify the effect of CUS on proliferation of DG progenitor cells, the same rats were injected with chlorodeoxyuridine (CldU, Sigma-Aldrich, MO, USA, 42.5 mg/kg, i.p.) 2 hours prior to sacrifice [17].

### Radial Arm Water Maze (RAWM)

The day after CUS exposure, rats (control, n = 15; stress, n = 15) were tested for spatial learning and memory performance using a one-day learning paradigm in the RAWM [18], which is a hippocampal-dependent task [19], [20]. The RAWM consists of six stainless steel, V-shaped arms inserted into a circular black pool filled with room temperature water made opaque with non-toxic paint. Rats were given 12 1-minute trials to find the “goal arm” where an escape platform was located 1 cm below the surface [21]. Available extra-maze visual cues included variously shaped figures on the walls. For each trial, animals were gently placed in the entrance arm facing the wall of the pool. Starting location arms for each trial were randomized, but never included the goal arm, which remained the same throughout all trials. If the rat could not find the platform within 1 minute, it was guided to and allowed to sit on the platform during the intertrial interval. During the 1-minute intertrial interval, animals remained on the platform. The 12 acquisition trials were divided into two blocks of six consecutive trials, interspersed with a 5-minute break. Following the acquisition trials, the animals underwent a short-term memory trial (30 minutes later) and a long-term memory trial (24 hours later). For each trial, latency to locate the platform and number of errors were recorded. Errors were operationally defined as anytime the animal’s entire body entered an arm that was not the goal arm, as well as anytime an animal entered the goal arm but did not find the hidden platform.

### Corticosterone Assessment

To verify that CUS and learning experience were stressful, we assessed corticosterone levels, using fecal boli, since they can be obtained without stress to the animal and fecal corticosterone is highly correlated with serum corticosterone [22], [23]. Fecal boli were collected from 12 randomly selected animals that experienced learning in the RAWM (control, n = 6; stress, n = 6). Baseline levels of corticosterone were determined from samples collected after animals had acclimated to their environment for a week but before CUS commenced. In order to see what impact CUS and the RAWM had on corticosterone, fecal samples were collected 24 hours after the last stressor and again following the long-term memory trial for the RAWM. Corticosterone levels were quantified using a commercially available Enzyme Immunoassay Kit (Assay Designs, Michigan, USA), according to the manufacturer’s instructions.

### Histology

One day after the end of CUS, control (n = 9) and stress (n = 9) animals were overdosed with anesthetic and intracardially perfused with 4% paraformaldehyde. Brains were removed and post-fixed overnight, then stored in 30% sucrose. Brains were cut into 50 µm sections on a freezing microtome and stored in cryoprotectant in 96-well microtiter plates at −20°C.

To label doublecortin-positive (DCX+) cells, standard immunohistochemical procedures were used to process every sixth section throughout the rostrocaudal extent of the hippocampus. Following treatment in 0.6% hydrogen peroxide and blocking in 3% donkey serum, sections were incubated for 72 hours at 4°C in primary antibody (goat anti-DCX, Santa Cruz Biotechnology, Inc., CA, USA, 1∶100), rinsed and then incubated overnight in secondary antibody (donkey anti-goat, Jackson ImmunoResearch, PA, USA, 1∶250). Sections were then processed with a standard ABC kit, and reacted in DAB according to the manufacturer’s instructions (Vector Labs, CA, USA). Sections were counterstained in methyl green, mounted onto slides and coverslipped.

For CldU and IdU immunohistochemistry, we followed the methods of Vega and Peterson [17]. Separate 1-in-6 series of sections were pre-treated in 0.3% hydrogen peroxide, rinsed in TBS, and then incubated in 2N HCl at 37°C for 10 minutes. Sections were then washed in 0.1M borate buffer for 10 minutes and rinsed six times in TBS. Thereafter, they were treated as described above. The antibodies used were mouse anti-BrdU (Becton Dickenson, NJ, USA, 1∶100) and rat anti-BrdU (Accurate Chemical, NY, USA, 1∶250) for CldU and IdU respectively. The secondary antibodies used were donkey anti-goat and donkey anti-rat (both Jackson ImmunoResearch, PA, USA, 1∶250).

### Stereology

To quantify IdU+ (surviving cells), CldU+ (proliferating cells) and DCX+ (new neurons) in the dorsal and ventral hippocampus, the optical fractionator probe was applied using our automated stereology system (StereoInvestigator, VT, USA). The average mounted section thickness was approximately 37 µm, so top and bottom guard zones were set at 5 µm each, for an optical dissector height of 27 µm. The dentate gyrus was traced at 10X, and then a grid of two-dimensional counting frames overlaid. The grid size was 60 x 60 and the counting frame size 40 x 40 [24]–[26]. For hippocampal subregions, the dorsal and ventral portions were separately quantified for IdU+, CldU+ and DCX+ somata, beginning at bregma −1.88 and ending at bregma −4.30 and beginning at bregma −4.52 and ending at bregma −6.04 for dorsal and ventral respectively [9], [27].

### Western Blotting

To generate a profile of region-specific expression of plasticity-associated proteins induced by a stressful spatial learning task, control (n = 7) and control+learning (n = 6) animals were sacrificed following the long-term memory trial. Brains were removed and the hippocampus rapidly dissected into 3 sections: dorsal, ventral and middle. A middle area was discarded in order to ensure that samples from the dorsal and ventral portions did not overlap [28]. The DG was then dissected away from the rest of the hippocampus, and the tissue was homogenized separately in 200 µl of lysis buffer cocktail (150 mM NaCl, 10 mM HEPES, 10 nM EGTA) and supplemented with 100x protease and phosphatase inhibitors (ThermoScience, IL, USA) with a sonicator at medium speed for 5 seconds, 4 times. The homogenates were then centrifuged at 14,000g for 15 minutes at 4°C. The supernatant was removed and stored at −80°C. The total protein concentration was estimated using a bicinchoninic (BCA) assay (Pierce Chemical, IL, USA) according to manufacturer instructions, using ß-actin as the standard.

Homogenates were separated on 17% SDS/PAGE gels (pro and mature BDNF) or 10% SDS/PAGE gels (PSD-95) (BioRad, CA, USA). They were then transferred onto a PVDF membrane for 1.5 hours at 45 V at room temperature (pro and mature BDNF), or overnight at 40 V at 4° (PSD-95), then stained with Ponceau S. Once rinsed, membranes were blocked for an hour at room temperature with continual mixing using 5% skim milk in TBS with 0.05% Tween-20 (BDNF, PSD-95) and 5% skim milk in PBS with 0.05% Tween-20 (ß-actin). Membranes were then washed 3 times for 5 minutes in wash buffer (TBS with 0.05% Tween for pro and mature BDNF and PSD-95; PBS with 0.05% Tween for ß-actin). Samples were incubated in primary antibody (polyclonal rabbit anti-BDNF, 1∶1000; mouse anti-PSD-95, 1∶500, both Chemicon, CA, USA; polyclonal mouse anti-ß-actin, 1∶20,000, Millipore, MA, USA) overnight at 4°C. After being washed in the appropriate buffer, membranes were incubated with secondary antibody (goat anti-rabbit 1∶15,000 or goat anti-mouse, 1∶5000, both KPL, Maryland, USA). Blots were developed using an enhanced chemiluminescence detection method (ECL Plus, Buckinghamshire, UK). Band intensity was assessed using a BioRad Gel Doc Imaging System with Quantity One software (BioRad, CA, USA). Protein quantity was assessed from the adjusted band intensity using the volume rectangle analysis tools and linear regression methods. Each sample value was divided by the total protein loading value (the intensity of ß-actin) and local background subtracted. Samples were expressed as optical density and compared across conditions and timepoints.

### Statistical Analysis

Data were analyzed with SPSS Statistics 17.0 (IBM SPSS Statistics, IL, USA). Corticosterone levels and RAWM acquisition trials were analyzed using repeated measures ANOVA. Short-term and long-term memory trials were analyzed using a one-way ANOVA. Neuroanatomical, protein, and body weight data were analyzed using a 2×2 ANOVA (Condition×Subregion or Condition×Time, as appropriate). P values below 0.05 were deemed statistically significant. Tukey post hoc comparisons were conducted where necessary, with an adjusted alpha of 0.05/2.

## Results

### Chronic Unpredictable Stress and Exposure to the Radial Arm Water Maze were Both Stressful Experiences

**Figure 1:**
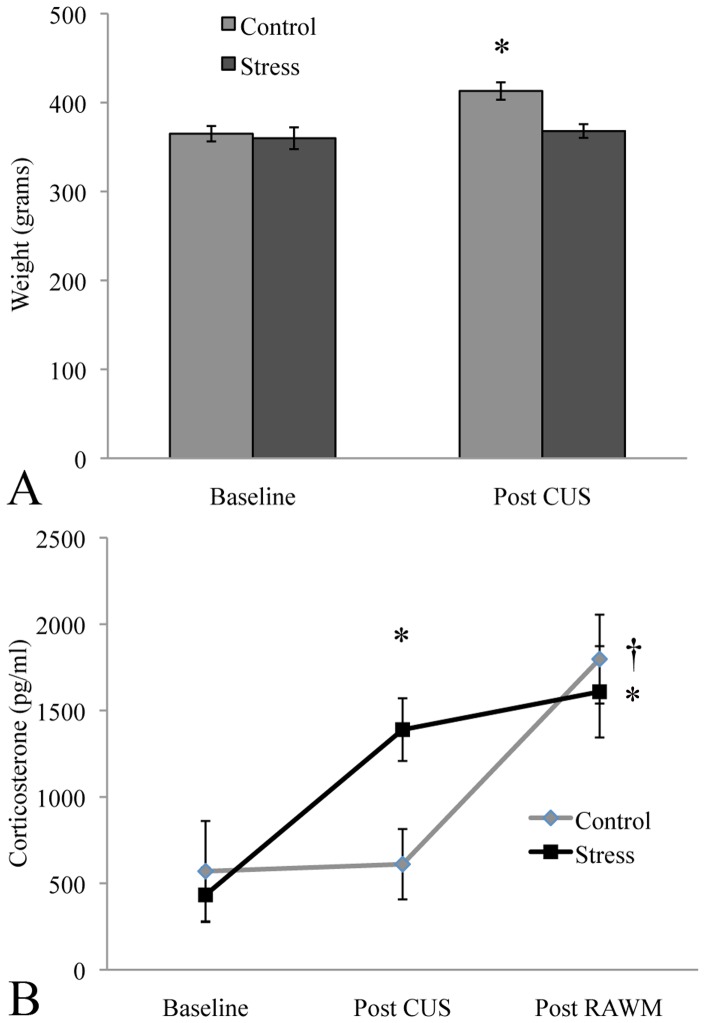
CUS and learning were both stressful. Animals that underwent CUS did not gain weight over the 2-week period of stressor exposure, whereas control animals did (A). Exposure to the CUS paradigm raised corticosterone levels, as did learning in the RAWM (B). Note, however, that learning did not further elevate corticosterone in stressed animals. *significantly different from baseline, † significantly different from Post CUS control.

Throughout the CUS paradigm body weights were monitored in both the control and stressed groups. Prior to onset of CUS, there was no difference in body weight between the groups. By the end of CUS, however, control animals had gained significantly more weight than stressed animals (see Figure 1A).

To determine whether CUS and learning experience were stressful to the animals, we assessed corticosterone levels. Fecal samples were collected from 12 randomly selected control and stressed rats that underwent the RAWM task. Control and stressed animals did not differ in corticosterone levels before onset of CUS (baseline). However, at the end of CUS, stressed animals had significantly higher corticosterone levels compared to controls, and had more than doubled their baseline levels. Corticosterone levels were significantly elevated in the controls by exposure to the RAWM to the point that they were no longer significantly different from CUS animals (see Figure 1B). CUS animals, however, did not show further elevation of corticosterone due to RAWM exposure.

### Chronic Unpredictable Stress Enhanced Long-term Spatial Memory

**Figure 2:**
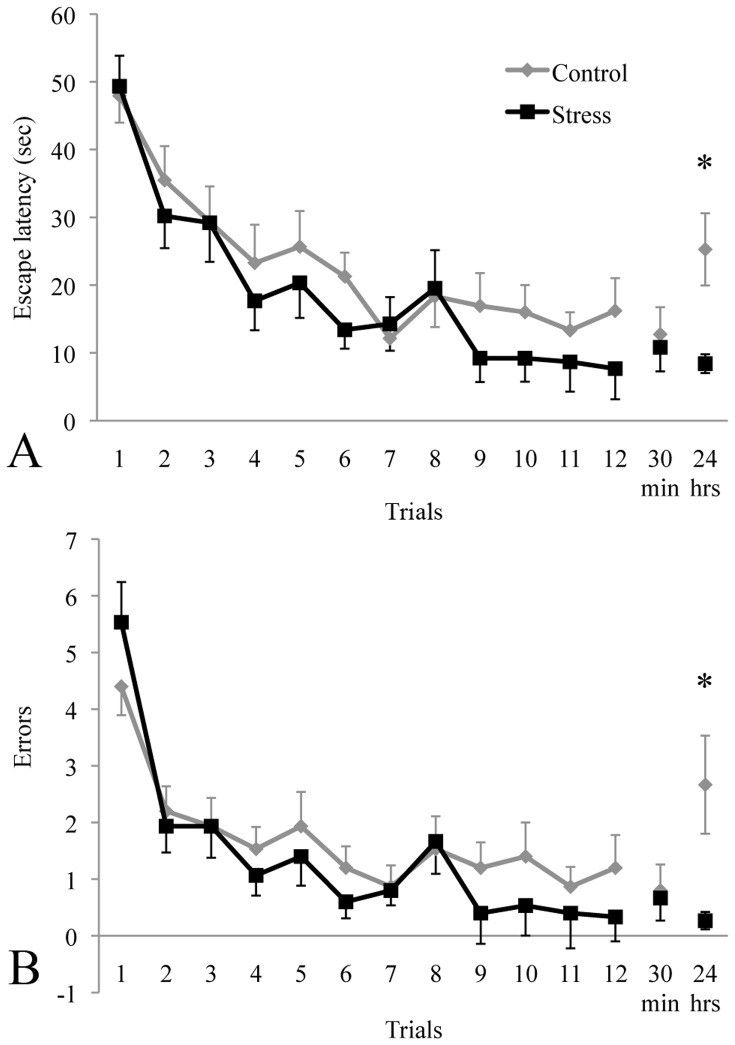
CUS facilitated long-term spatial memory in the RAWM. Escape latencies did not differ between control and stressed animals during the acquisition trials (1–12), or on the short-term memory trial (30 min) (A). However, stressed animals took significantly less time to locate the hidden platform on the long-term memory trial (24 hrs). A similar pattern was seen for errors made during search (B). * significantly different from control.

Following CUS, control and stressed animals were exposed to the RAWM to evaluate spatial learning and memory. There was no difference between groups in latency to find the hidden platform or number of errors made during the acquisition (trials 1–12) of the RAWM learning task (see Figure 2A–B). Furthermore, there was no significant difference between groups for latency or errors for the short-term memory trial. However, stressed animals found the platform significantly faster and made fewer errors in the long-term memory trial.

### Chronic Unpredictable Stress most Severely Affected Neurogenesis in the Ventral Dentate Gyrus

**Figure 3:**
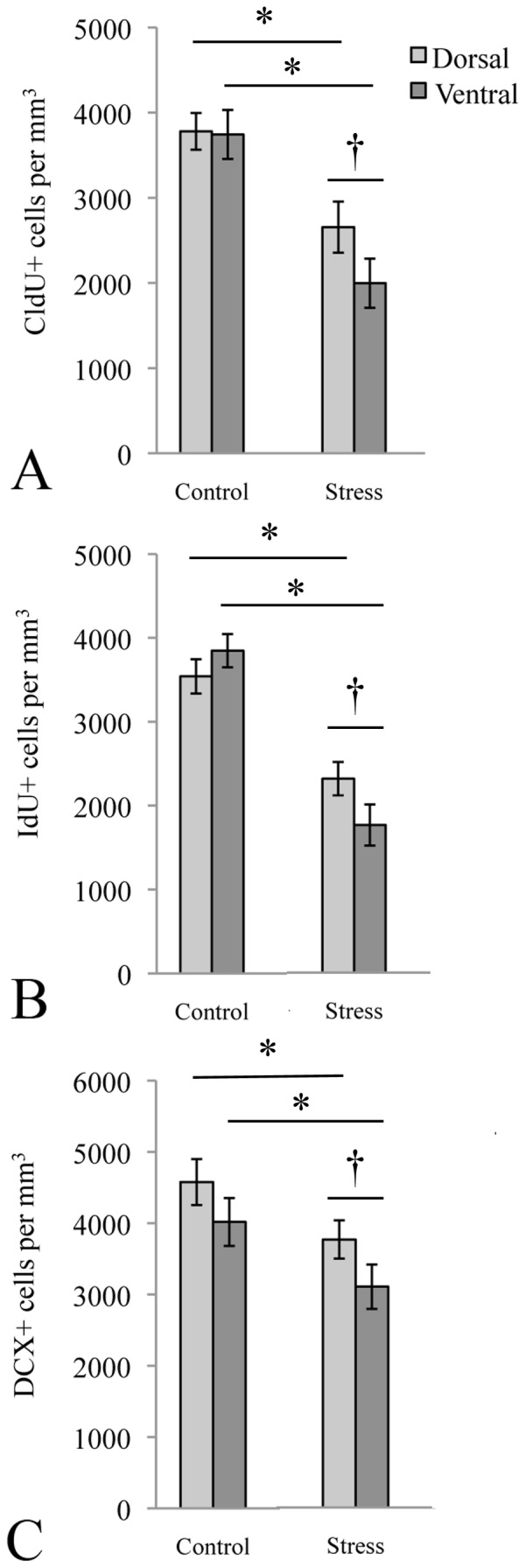
Stress most severely affected neurogenesis in the ventral dentate gyrus. Compared with controls, rats in the CUS group showed decreased proliferation (A), survival (B) and neuronal differentiation (C) in the dentate gyrus. This effect was most pronounced in the ventral, compared to the dorsal, sub-region († indicates significant difference between subregions). * significantly different from control.

To determine the effects of CUS on hippocampal neurogenesis, we stereologically quantified cell proliferation (CldU+ cells), survival (IdU+ cells) and neuronal differentiation (DCX+ cells) in the dorsal and ventral hippocampal subregions. A similar pattern was found for all 3 markers. Compared to control rats, CUS animals had significantly fewer CldU+, IdU+ and DCX+ cells in both subregions (see Figure 3). In addition, within the stressed condition, there were significantly fewer CldU+, IdU+ and DCX+ cells in the ventral subregion, compared to the dorsal, indicating that the ventral sub-region was worst affected by stressful experiences.

### A Stressful Learning Experience Altered Expression of Plasticity-associated Proteins in a Region-specific Manner

**Figure 4:**
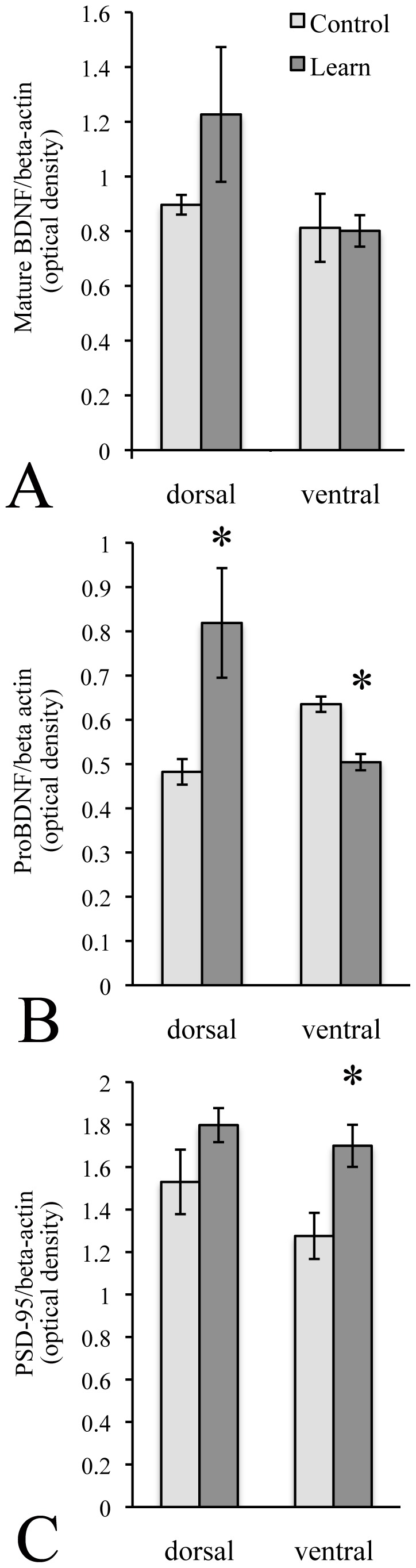
A stressful spatial navigation task differentially affected protein expression in the dorsal and ventral subregions. Expression of mature BDNF was not significantly changed by RAWM exposure in either the dorsal or ventral dentate gyrus (A). In contrast, proBDNF was significantly increased in the dorsal dentate, and significantly decreased in the ventral (C). PSD-95 was unchanged in the dorsal, but significantly increased in the ventral dentate (C). * significantly different from control.

In order to determine whether an experience that was both stressful and involved spatial navigation would differentially affect protein expression in the dorsal and ventral DG sub-regions, Western blotting was used to quantify expression of mature BDNF, its precursor proBDNF and the synaptic scaffolding protein, PSD-95. Rats were sacrificed after completion of the long-term memory trial in the RAWM. One dorsal sample from a control animal was omitted because there was too little protein to be detected. For BDNF, there were no significant differences between groups in either the dorsal or ventral subregions (see Figure 4A). However, RAWM experience significantly increased proBDNF in the dorsal sub-region, and significantly decreased it in the ventral (see Figure 4B). RAWM experience did not change PSD-95 expression in the dorsal DG, but significantly elevated it in the ventral (see Figure 4C).

## Discussion

### Chronic Unpredictable Stress Enhanced Long-term Spatial Memory

Although the entire hippocampus is stress-sensitive, the ventral portion appears selectively vulnerable to the negative effects [7], [8]. We have previously shown that neuroadaptive responses to CUS, including expression of NPY and ΔFosB, are more pronounced in the dorsal hippocampal subregion [9]. Because this subregion has been implicated in spatial function [4], [29], [30], we reasoned that stress-induced plasticity there might confer an advantage in a spatial task. Therefore, in the present study, we compared the performance of animals that had been through a 2-week paradigm of CUS to that of stress-naïve animals using a one-day learning paradigm in the RAWM [21]. Our results show that although there was no difference between groups in acquisition or short-term memory trials, animals that underwent 2 weeks of CUS had better long-term memory for platform location.

Although stressors increase corticosterone, which has damaging effects on the brain (see [6] for review), a large literature attests to the idea that stress does not necessarily detract from learning, and may even enhance it. Indeed, a great many variables influence this relationship, such as the type of stress and the type and difficulty of the learning task (see [31] for review).

In the case of spatial learning, adaptive stress-induced plasticity in the dorsal hippocampus may preserve or enhance learning and other adaptive responses. The results of the present study, including enhanced long-term spatial memory, and the lack of any stress-induced decrement in performance during acquisition trials, suggests that the dorsal hippocampus may be stress-resilient, resulting in preserved, or even enhanced capacity to make adaptive responses.

### Chronic Unpredictable Stress most Severely Affected Neurogenesis in the Ventral Subregion

We have previously shown that survival of newborn cells was better preserved in the dorsal dentate (compared to the ventral) following CUS [9]. In the present study, we used stereology to quantify proliferating cells labeled by CldU 2 hours prior to sacrifice, and surviving cells labeled by IdU during the first five days of the CUS paradigm. We found that CUS decreased the number of CldU+ cells in both the dorsal and ventral subregions of stressed animals. The decrease was greatest in the ventral subregion. The same pattern was found for IdU+ cells.

We also quantified the number of DCX+ cells in both sub-regions. Again, although CUS decreased DCX+ cells in both sub-regions, there were significantly fewer DCX+ cells in the ventral subregion of stressed animals. Taken together, these results suggest that although neurogenesis in both hippocampal subregions is negatively affected by chronic stress, the dorsal subregion may be more resilient. Relatively better preservation of neurogenesis in the dorsal subregion may provide a substrate for spatial learning in a stressful situation, thereby maintaining the potential for escape.

### A Stressful Learning Experience Differentially Affected Expression of Plasticity-associated Proteins in the Hippocampal Subregions

The hippocampus is a structurally and functionally complex area of the mammalian brain. Although its roles in two major functions, spatial navigation and emotional responses, have been well-established, they are usually examined separately. However, stressful situations may involve the need for spatial navigation, and, conversely, spatial navigation tasks can be stressful. Therefore, we set out to quantify the expression of plasticity-related proteins in the dorsal and ventral subregions of the hippocampus in response to a situation that simultaneously tapped the functions of both – learning in the RAWM. Although rats are excellent swimmers, they are stressed by exposure to water, therefore learning tasks that involve swimming are stressful for them [10]. We examined the neuroplastic responses of the two subregions following learning in the RAWM by quantifying the expression of BDNF, its immature isoform (proBDNF) and the synaptic scaffolding protein PSD-95.

BDNF was elevated in the dorsal dentate by learning in the RAWM, but not significantly. It has been shown previously that BDNF levels are elevated in the hippocampus for up to 12 hours after learning [32]–[34] but returned to baseline levels by 24 hours post-learning [34]. In the present study, animals were sacrificed immediately following the RAWM long-term memory trial, which was 24 hours after the final RAWM acquisition trial. Thus, BDNF may have been upregulated more immediately after acquisition trials, and the recent exposure to a single memory trial was not sufficient to significantly re-increase expression.

BDNF is formed from cleavage of its precursor, proBDNF, a biologically active intermediate that may contribute to long-term depression and other effects counter to those of BDNF [35], [36]. In the present study, proBDNF was significantly elevated in the dorsal dentate gyrus 24 hours after the acquisition trials in animals that learned. While it is possible that proBDNF elevation may be exerting independent effects, its conversion to BDNF has been shown to occur in an activity-dependent manner [35]. Thus, it may be that the observed increase in proBDNF is an indication that it is being increasingly converted to mature protein for immediate use. Moreover, assuming it was rapidly converted to BDNF, the preferential increase in proBDNF in the dorsal subregion may in part underlie the superior RAWM performance of animals that underwent CUS. In contrast to the dorsal increase, proBDNF was significantly decreased in the ventral dentate gyrus, providing further evidence that stressful situations more adversely affect the ventral (compared to the dorsal) hippocampus.

PSD-95, also known as SAP-90, is a protein that is a member of the membrane-associated guanylate kinase (MAGUK) family. It is almost exclusively located at the post-synaptic density of neurons [37], and is involved in the anchoring of synaptic proteins like neuroligin, potassium channels, AMPA receptors and NMDA receptors [38]. In the present study, PSD-95 was significantly elevated in the ventral, but not the dorsal subregion of the dentate gyrus. This suggests that the emotional component of the learning task (the stress associated with performing the water maze task) selectively altered synaptic structure in the ventral subregion. Interestingly, in the present study there was an increase in proBDNF in the dorsal hippocampus, and a trend towards an increase in mature BDNF, but this did not result in an increase in PSD-95, even though increasing levels of BDNF can increase PSD-95 in spines [39]. This suggests that BDNF’s role in this learning situation is to act as a signaling molecule involved in facilitating changes in synaptic efficacy [40], [41] rather than synaptic structure [42]. Although there may be alternative explanations, it is clear that in the present study there was a dissociation between changes in the levels of pro and mature BDNF and PSD-95 expression in animals exposed to the RAWM.

### Conclusions

In the present study, we found that chronic unpredictable stress enhanced spatial memory. We also showed that chronic unpredictable stress impacted neurogenesis more severely in the ventral component of the dentate, compared to the dorsal, suggesting that the dorsal component may be more stress-resistant. Finally, we showed that a situation that draws simultaneously on the established functions of both the dorsal (spatial navigation) and ventral (emotional responses) hippocampal subregions differentially affects protein expression in those areas. Taken together, these data uphold the notion that the hippocampus plays a dual role in the response to stress. The more stress-resilient dorsal portion may be involved in behavioral adaptations, such as escape from or neutralization of the stressor, whereas the ventral portion may be more involved in emotional responses.
